# MPLA Case 1: Implementing Cone‐Beam CT in a Community Hospital

**DOI:** 10.1002/acm2.13185

**Published:** 2021-03-19

**Authors:** Dongxu Wang, Gabbie Meis, William Ellet, Leonard Kim, Cassandra Stambaugh, Mary Gronberg, Jennifer Johnson

**Affiliations:** ^1^ Memorial Sloan Kettering Cancer Center New York NY USA; ^2^ The University of Iowa Iowa City IA USA; ^3^ Miami School of Business University of Miami Coral Gables FL USA; ^4^ MD Anderson Cancer Center at Cooper Camden NJ USA; ^5^ Tufts Medical Center Boston MA USA; ^6^ University of Texas MD Anderson Cancer Center Houston TX USA; ^7^ Kelsey‐Seybold Clinic Houston TX USA

**Keywords:** MPLA, leadership, case study

## Abstract

This fictional case describes a managerial situation of implementing cone‐beam computed tomography faced by a solo medical physicist in a rural community hospital. The intended use of the case study, in either a facilitated learning session or self‐study, is to inspire the readers to discuss the situation, analyze the institutional and personal factors, apply relevant leadership skills, and propose action plans. This case study falls under the scope of, and is supported by, the Medical Physics Leadership Academy (MPLA). A sample facilitator’s guide or self‐study guide is included in the manuscript for reference by users of this case study.

Dr. Jessica Garner had been working at Concord Hospital^1^ for two months and was having trouble adjusting. The patients, doctors, and staff she worked with welcomed her into their community with open arms. She had been born and raised in Concord, MT, and returning to it with her family was in every sense of the word a homecoming. But her years of professional training in San Francisco had left an indelible mark on her, and she was having a hard time accepting the medical technology limitations she now faced.

Dr. Garner had chosen Concord, MT over a handful of open positions. After visiting the area for her interview, she knew this would be the place where she would be most comfortable settling down and raising her two young boys, and her husband agreed. A postinterview stop at the local grocery store offered held doors and warm smiles as she picked up snacks and drinks for the long drive back to the Bay Area. She would be the first full‐time, hospital‐employed medical physicist at Concord, and she was determined to use her academic background for good. She appreciated that the hospital had decided to hire a full‐time staff medical physicist instead of relying on a physics consulting company that sent in different people once a week.

Dr. Garner was eager to implement the cone‐beam computed tomography (CBCT) on the Varian 21EX Linear Accelerator (linac). The Varian 21EX was a staple in radiation therapy treatment in the hospital’s cancer clinic. She knew the standard of care of three‐dimensional image guided radiation therapy (IGRT) with CBCT could easily be delivered by the Varian 21EX equipped with the on‐board imager. In her office Dr. Garner sat down in the comfortable nylon chair the hospital had purchased for her. She used a disinfectant wipe to clear off the thin layer of dust that had collected along the top of the computer monitor and on its stand.

She had just finished the CBCT acquisition of a phantom on the linac. Everything worked fine so far. Now she was ready to load the CBCT in MOSAIQ on an office workstation. She clicked on the “Review” button, and there was no response. She waited, knowing the computer was significantly slower than the GPU‐equipped one she had used at her previous employer, a high‐ranking research hospital in San Francisco. At Concord Hospital, she was a little disappointed that the computer workstation assigned to her had low specifications. She was told it met the hospital’s IT specifications, and everybody else had the same computer.

Five minutes passed and the CBCT she had acquired finally showed up in MOSAIQ. However, she couldn't scroll the image slices, move, or zoom. The image on the screen froze. She gave up and pressed “Ctrl+Alt+Dlt.”

At Dr. Garner's previous position, CBCT had been common and was the standard method for image‐guided radiation therapy. She was puzzled that this hospital, which had the foresight to hire a full‐time medical physicist, used computers with such poor performance. Not utilizing CBCT due to computer performance had to be a mistake. She decided to check with Mark Robinson, the on‐site medical dosimetrist, who had worked here through many generations of technologies.

Dr. Garner found Mark upstairs in his small office, patiently waiting for a plan optimization to finish. Not wanting to startle him, Dr. Garner rapped her knuckles along the doorframe to announce her arrival. After they exchanged greetings, Dr. Garner asked whether there had been problems with CBCT review in MOSAIQ.

Mark sighed. "It’s been like this forever. We’ve never used it successfully. We tried a few times before, but the computers couldn’t handle it.”

“Isn’t there something we can do to fix that? A simple computer update or a new computer for the system? CBCT is commonplace. It’s strange that it’s not being used here.”

Mark nodded, but his facial expression conveyed resignation. “I’ve been here for 18 years, and we seem to always have low‐end computers for office work.”

“But this isn't office work. It's high‐tech medical work!”

“I completely agree!” Mark said. “Maybe you can change this. If you’d like, we can put in a request for new equipment, but the clinical supervisor is wary of any additional investments in computers, and the IT department has specific requirements about which programs we’re allowed to run. With those restrictions, I’m not sure if we’ll be able to do much. But maybe you'll make the difference. They hired you to do the high‐tech work in the first place.”

Dr. Garner couldn't help noticing that his tone of voice didn't sound hopeful.

Concord Hospital’s junior radiation oncologist, Dr. Aaron Mitchell, had joined the practice a year before Dr. Garner. He finished his residency in the same research hospital as Dr. Garner and felt lucky to be given the opportunity to practice only a few hundred miles from where he grew up, where job opportunities for radiation oncologists were sparse. In the past year, he had not only convinced the hospital to hire a full‐time medical physicist but was also able to recruit Dr. Garner, his residency physics mentor in San Francisco.

However, Dr. Mitchell was beginning to wonder if he had settled for the easiest option. The equipment he worked with felt clunky in comparison to the first‐rate machines at his previous hospital. He had turned down an offer at his resident hospital, wanting to slow things down a bit to return to his family. After a year in Concord, Dr. Mitchell was disappointed and found himself in frequent disagreement with his senior practicing partner, Dr. David Bell.

Dr. Bell’s treatment methods were straightforward, reflecting his 30+ years of practice, but Dr. Mitchell wanted to be innovative and try new treatment methods he learned during residency. However, the technology in Concord did not always allow that, and on other occasions, Dr. Bell shot him down with a stock comment: “Now, that’s not how we do things here.” He knew Dr. Bell had many more years of experience than he did but felt Dr. Bell was simply waiting to retire, going through the motions of practice and assigning the same treatments he had for years.

Dr. Mitchell desired to get along with his senior partner, but he didn’t want to become complacent. It came down to what was best for his patients, and he knew he could and should do better by them. He checked his watch and realized he had two minutes to make it to Dr. Bell’s office for their consultation with Dr. Garner.

Dr. Bell had just sat down in his office chair, the old, worn‐out springs creaking into position. Dr. Mitchell would send him email after email with articles from medical journals about new methods, despite his request for printed copies of the articles. Dr. Garner cluttered Dr. Bell’s inbox with new ideas for equipment and technology, occasionally citing her own research in San Francisco. Dr. Bell typically read a few of the articles Dr. Mitchell emailed to him. The studies often came from hospitals with more impressive equipment than Concord could ever hope to afford. His retirement was not far off. His younger colleagues would soon have their turn.

The two young colleagues now came to his office for their biweekly meeting. Dr. Garner carried her laptop and a thin notebook, while Dr. Mitchell had brought a pocket‐sized, leather‐bound book in which he would take notes.

Dr. Garner began the conversation. “The Varian 21EX is not working at its full potential‐‐we’re not using cone‐beam computed tomography.”

“Well, is that important?” said Dr. Bell.

“I’d say so," said Dr. Garner. "AAPM has produced a survey showing that CBCT is the standard practice for IGRT. It’s not being used here because our computers are too slow. They freeze or crash before we can review the results.”

Dr. Mitchell barely resisted the urge to roll his eyes. Of course, the computers were too old to run something of that caliber. They were probably less expensive than his own ergonomic chair. “If we’re not administering CBCT," he said, "we’re not capturing the charges related to CBCT. I think the additional revenue would easily cover the cost of purchasing new computers.”

"You're up on the research, I assume. CBCT leads to better treatment outcomes?"

There was an awkward silence that Dr. Garner broke. "There isn't definitive research evidence that it does, at least not yet. But we're sure it's a step in the right direction. In San Francisco, the doctors and patients preferred the upgrade."

Dr. Bell nodded. "OK, but I read in an article you sent me that CBCT is only useful for 40 percent of cancer cases and will improve results in a small percentage of the 40 percent. Can you prove that the cost is worth the benefit here?"

"That's a fair question," said Dr. Mitchell.

"Shouldn't we let the deep‐pocketed treatment centers figure this out before we plunge ahead and spend dollars we don't have?"

"I don't think the costs are going to be prohibitive for us, and we could be saving lives that would be lost otherwise," said Dr. Garner. "It might be just two or three a year, but in a community this size, that makes a big difference."

No one said anything for a while.

“I don't want to seem cold hearted," said Dr. Bell. "But won't our therapists and dosimetrist have to be trained on the new equipment? That's going to be a significant cost for the hospital. I've seen it many times before. The implementation of new technology is much harder and more expensive than the vendor says it will be."

“I believe it’s what’s best for the hospital and for the patients,” Dr. Garner answered. “You can have better than 2‐mm accuracy in target alignment with CBCT!”

“I say go for it,” said Dr. Mitchell.

Dr. Bell shrugged in response. “I think what we have works fine, but if you two agree, I don't want to stand in the way. I hope you're ready for a fight, though.” Although he would never admit it to his younger colleagues, Dr. Bell wasn’t especially comfortable with technology and even struggled with his office computer.

Dr. Mitchell and Dr. Garner spoke at almost the same time, "What do you mean?"

Dr. Bell looked serious. "IT isn't going to give in on expensive new computers easily. They'll be afraid that every practice in the hospital will want one. Administration will just see the cost side of upgrades and training‐‐and mistakes. They'll see you as hotshot doctors from California who want the best, whatever it costs."

Dr. Bell paused for a moment. "This is on you‐‐I'm not leading the charge. And I've got to give you fair warning that you'll have to make it very simple for old folks like me to use this new technology. Otherwise, I can't promise I'll prescribe it.”

Dr. Garner returned to her office to think about the conversation. She genuinely believed that using CBCT for IGRT was better for patient care and treatment outcomes. What can she do?

## AUTHOR CONTRIBUTIONS

DW and GM drafted and revised the case text. WE and JJ made critical revision of the case text. DW, LK, CS, and MG drafted and revised the sample facilitator’s guide. All authors have approved the final version.

## Note

^1^ Concord, MT is intended to be representative of a rural community hospital. Any association with actual practices anywhere is purely coincidental and unintentional.

## SAMPLE FACILITATOR’S GUIDE OR SELF‐STUDY GUIDE FOR MPLA CASE 1

### 1 Case overview

After having worked many years at a large academic hospital, Dr. Garner returns to her hometown for a clinical medical physicist position at a community hospital. She enjoys the slower pace of the small town and feels that it is a great place to raise her kids. Shortly upon arrival, Dr. Garner realizes that the hospital is not using cone‐beam computed tomography (CBCT) on their linear accelerator, which is the standard of care for three‐dimensional image guided radiation therapy (IGRT). She learns that the department’s computers are not powerful enough to process CBCT images.

Dr. Garner and her colleague Dr. Mitchell, a junior radiation oncologist, confront the senior practicing physician, Dr. Bell. Dr. Bell explains that as a small clinic with limited budget, the costs of upgrading the computers and training personnel is prohibitive. He asks his young colleagues of any evidence of better clinical outcomes to support the need for CBCT, to which they cannot answer. Dr. Bell warns them of the hurdles they will face with IT and administration if they decide to move forward.

This case is a *Decision Scenario*: the case presents a “need to make a critical decision and potentially persuade” others to accept it^1^.

### 2 Learning objectives

1. Recognize the impact that culture has on clinical practice.
2. Prepare an action plan to sell a new idea to members of the clinic with different personalities and incentives.
3. Understand that cost‐benefit analysis or return‐on‐investment are often the key metrics to convince hospital administration to adopt new technology, which may bring in high‐quality care as well as new revenues.

### 3 Pedagogy

- **Audience:** medical physics and radiation oncology students, residents, and faculty
- **Prerequisites:** Basic understanding of image‐guided radiation therapy and cone‐beam computed tomography
- **Supplemental materials:** Kotter’s 8‐Step Change Model^2^

### 4 Discussion questions

1. Does this case resonate with you and some of the challenges that you face?
2. What does the situation look like from Dr. Bell’s point of view?
3. What does the situation look like from Dr. Garner’s point of view?
4. How does Dr. Bell and Dr. Garner’s previous work culture impact their interaction with each other?
5. Should Dr. Garner try to persuade Dr. Bell?
6. What are three things that Dr. Garner can do to help persuade Dr. Bell of the need for change and to get his support?
7. What other obstacles should Dr. Garner prepare for?
8. At one point, Dr. Mitchell mentioned that IGRT charges enabled by CBCT might cover the computer cost. What resources can you look into to come up with such a cost‐benefit analysis or return‐on‐investment analysis?

### 5 **Case analysis** (sample answers to discussion questions)

#### 5.1 Remember that there are no correct answers!

1. While not everyone has experienced this exact scenario, they likely have experienced something similar. Compare the challenges that the group has faced and summarize common themes.
2. Dr. Bell’s point of view:
  - Dr. Bell is aware of the hospital culture and budget and has experienced the challenges that may come with practice transition. He may be averse to facing those challenges again.
  - Dr. Bell values practicality and evidence versus the latest and greatest technology.
3. Dr. Garner’s point of view:
  - Dr. Garner is new to the clinic and is adjusting from a large and well‐funded institution to a community hospital with limited resources and staff.
  - She is less familiar with the hospital budget.
  - She lacks experience with business proposals.
4. Dr. Garner is accustomed to a culture of support where projects are completed quickly with little adversity due to working in an institution with both vast funding and personnel. Dr. Bell, alternatively, is in a culture of adversity and has been faced with many obstacles that he does not feel that he has any control over in regards to new technology and processes due to lack of resources, both with personnel and technology. The different histories of cultures of support v.s. culture of adversity without taking the time to understand different perspectives automatically creates tense interactions with feelings of misunderstanding.
5. Pro: Dr. Garner should continue trying to persuade Dr. Bell.
  - The clinic has already invested a lot of money into purchasing CBCT. The expense of upgrading the computers and training personnel is small compared to the cost of CBCT.
Against: Dr. Garner should listen to Dr. Bell and stop pushing for CBCT.
- Dr. Garner is new to the clinic. She should prioritize building relationships before trying to make changes.
Win/Win (or Neutral): Dr. Garner should use this opportunity to build a working relationship with Dr. Bell.
  - Dr. Garner should acknowledge his concerns, research solutions and present options that both meet Dr. Bell where he is and appropriately utilize the technological tools that the hospital has invested in. This would lay the groundwork for future projects and create a collegial environment instead of an adversarial one.
6. Example action items/ path forward.
  - Find shareable middle ground and start small. For example, Dr. Garner could propose that the computers at the treatment console be upgraded first.
  - Develop a business strategy/financial case that addresses cost and revenue.
  - Present evidence of better clinical outcomes with CBCT or examples where CBCT would make a difference in clinical outcomes and safety (for example, being able to use tighter margins).
  - Enable Dr. Bell, the dosimetrist and therapists to experience CBCT technology through vendor presentation or shadowing at a neighboring hospital.
  - If and when middle ground is reached, prepare a project plan that encompasses the needs and goals of the department surrounding this upgrade in technology.
7. Other potential obstacles:
Administration:
  - Since additional resources may be required to implement CBCT technology, Dr. Garner should be prepared to present the cost of the project as well as the added benefit to the hospital administration to obtain appropriate funding.
IT:
  - Dr. Garner should develop a relationship with the IT department to educate herself on the infrastructure challenges that are faced in her clinic. She should work with IT to brainstorm solutions to meet the needs of the department.
Radiation Therapists:
  - Dr. Garner should engage with the lead radiation therapist to ensure that the needs of the radiation therapists are being considered as part of this change. Training will also need to be arranged so that the therapists know how to use the new technology.
Dosimetry:
  - Dr. Garner should ensure that the dosimetry staff is consulted and trained on the additional plan preparation needed to support using CBCT for image guidance.
Ongoing education:
  - Dr. Garner should anticipate that there will be growing pains associated with the introduction of new technology. Appropriate policies, procedures and protocols should be put into place and resources for refresher education for the new technology should be budgeted into any project plan.
8. A great resource for learning and understanding radiation therapy billing is AAPM’s Professional Economic Committee. ASTRO also publishes annual coding guide and billing refresher. To come up with an accurate financial projection *pro forma*, collaboration with the hospital billing staff is needed. For learning the subjects of organizational finance and budgeting, a useful resource is Harvard ManageMentor^3^ (https://hbr.org/harvardmanagementor/, paid subscription required).

### 6 Teaching plan

1. Read the case‐ 15 min.
2. Discussion Questions‐ 45 min..
3. Teach the 8 step change model and apply it to the case‐ 30 min.

### 7 CONCLUSION

The students should leave the discussion with a greater understanding of the differing viewpoints of clinical staff. They should have a better appreciation for hospital budget, leadership structure, and building relationships. Lastly, they should be able to identify the necessary aspects for successful change management.
